# Supplementary material for: Hydrophobic Nanoporous Silver with ZIF Encapsulation for Nitrogen Reduction Electrocatalysis
Source: Molecules. 2023 Mar 20;28(6):2781. doi: 10.3390/molecules28062781 (PMC10051616; doi:10.3390/molecules28062781)
Supplement: Supplementary file 1 [file molecules-28-02781-s001.zip › molecules-2285255-supplementary.pdf]

Supplementary Materials

Hydrophobic Nanoporous Silver with ZIF Encapsulation for Nitrogen Reduction Electrocatalysis

Yating Qi, Shulin Zhao, Yue Pang and Yijie Yang *

Tianjin Key Laboratory of Structure and Performance for Functional Molecules, College of Chemistry, Tianjin Normal University, Tianjin 300387, China
* Correspondence: hxxyyyj@tjnu.edu.cn

Figure S1. TEM images of (a) NPS and (b) NPS@ZIF.

Figure S2. Characterization of NPS. (a) HAADF-STEM image of NPS and EDS mapping of elements (b) Ag, (c) Cl. (d) Corresponding EDS elemental spectrum.

Figure S3. XPS characterization of NPS.

Figure S4. IR spectra of NPS, ZIF and NPS@ZIF.

Figure S5. (a) Nitrogen sorption isotherms at 77 K. (b) Corresponding pore size distributions.

Figure S6. (a) Absorbance spectra of indophenol blue in NH_4^+ solutions at various concentrations. (b) Linear correlation of the absorbance intensity to NH_4^+ concentration.

Figure S7. (a) Absorbance spectra of N_2H_4 solutions with various concentrations after reacting for 20 min at room temperature. (b) Corresponding calibration curve. (c) Yield of ammonia and hydrazine generated during ENRR at -1.0 V vs. RHE.

Figure S8. TEM image of NPS@ZIF after long-term electrocatalysis.

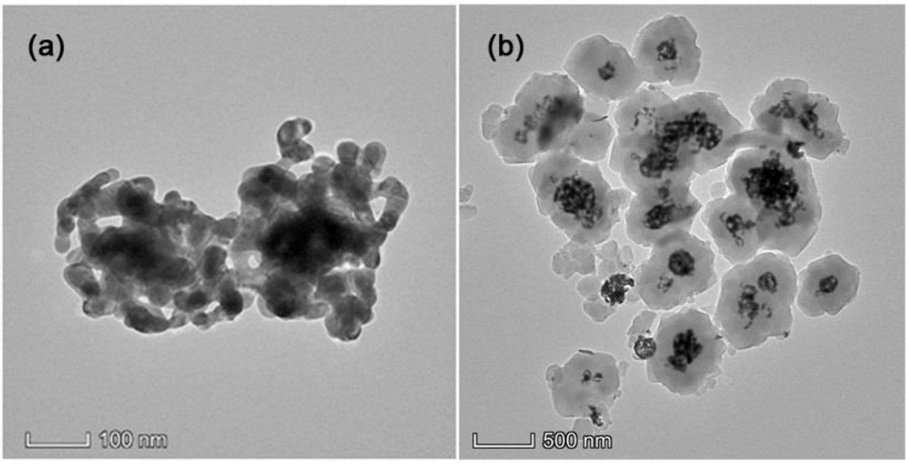

Figure S1. TEM images of (a) NPS and (b) NPS@ZIF.

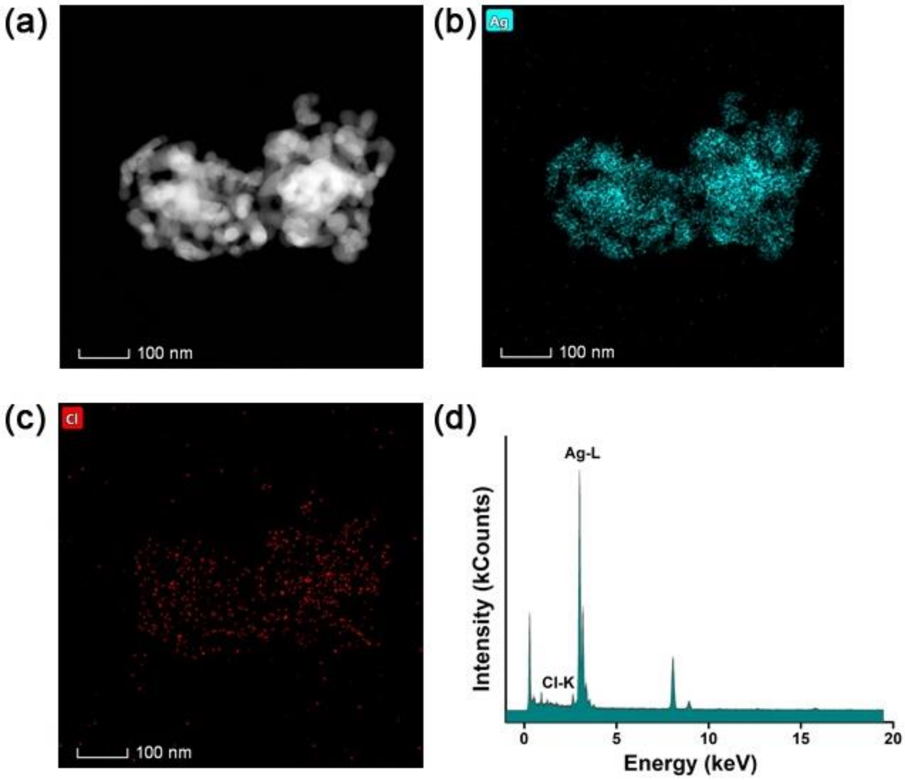

Figure S2. Characterization of NPS. (a) HAADF-STEM image of NPS and EDS mapping of elements (b) Ag, (c) Cl. (d) Corresponding EDS elemental spectrum.

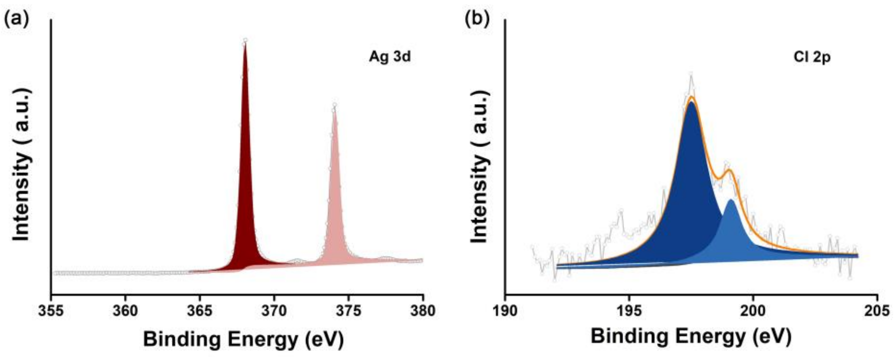

Figure S3. XPS characterization of NPS.

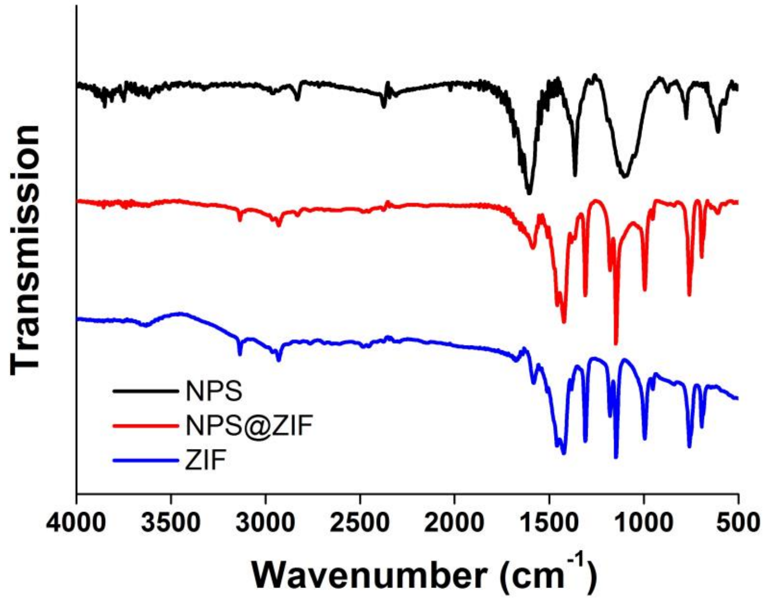

Figure S4. IR spectra of NPS, ZIF and NPS@ZIF.

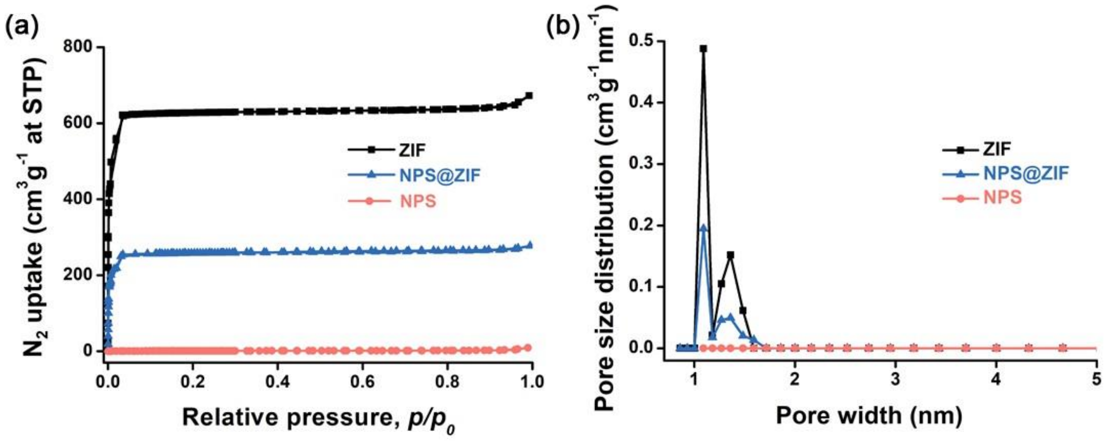

Figure S5. (a) Nitrogen sorption isotherms at 77 K. (b) Corresponding pore size distributions.

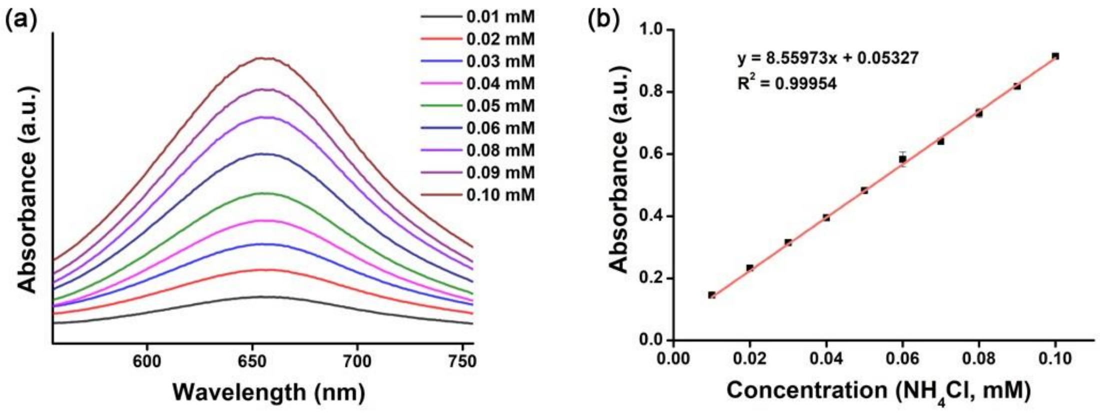

Figure S6. (a) Absorbance spectra of indophenol blue in NH_4^+ solutions at various concentrations. (b) Linear correlation of the absorbance intensity to NH_4^+ concentration.

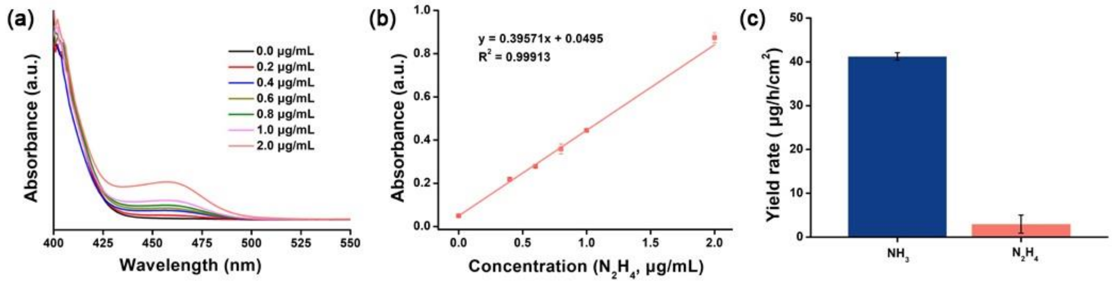

Figure S7. (a) Absorbance spectra of N_2H_4 solutions with various concentrations after reacting for 20 min at room temperature. (b) Corresponding calibration curve. (c) Yield of ammonia and hydrazine generated during ENRR at -1.0 V vs. RHE.

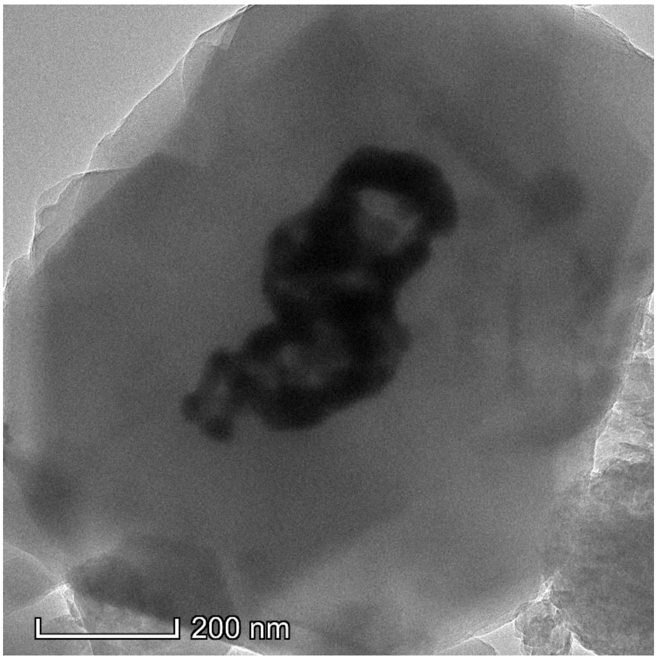

Figure S8. TEM image of NPS@ZIF after long-term electrocatalysis.
